# Staggered larval time-to-hatch and insecticide resistance in the major malaria vector *Anopheles gambiae *S form

**DOI:** 10.1186/1475-2875-9-360

**Published:** 2010-12-14

**Authors:** Maria L Kaiser, Lizette L Koekemoer, Maureen Coetzee, Richard H Hunt, Basil D Brooke

**Affiliations:** 1 Malaria Entomology Research Unit, School of Pathology, Faculty of Health Sciences, University of the Witwatersrand, Johannesburg, South Africa; 2 Vector Control Reference Unit, National Institute for Communicable Diseases of the National Health Laboratory Service, Private bag X4, Sandringham, 2131, South Africa; 3 School of Animal, Plant and Environmental Sciences, University of the Witwatersrand, Johannesburg, South Africa

## Abstract

**Background:**

*Anopheles gambiae *is a major vector of malaria in the West African region. Resistance to multiple insecticides has been recorded in *An. gambiae *S form in the Ahafo region of Ghana. A laboratory population (GAH) established using wild material from this locality has enabled a mechanistic characterization of each resistance phenotype as well as an analysis of another adaptive characteristic - staggered larval time-to-hatch.

**Methods:**

Individual egg batches obtained from wild caught females collected from Ghana and the Republic of the Congo were monitored for staggered larval time-to-hatch. In addition, early and late larval time-to-hatch sub-colonies were selected from GAH. These selected sub-colonies were cross-mated and their hybrid progeny were subsequently intercrossed and back-crossed to the parental strains. The insecticide susceptibilities of the GAH base colony and the time-to-hatch selected sub-colonies were quantified for four insecticide classes using insecticide bioassays. Resistance phenotypes were mechanistically characterized using insecticide-synergist bioassays and diagnostic molecular assays for known reduced target-site sensitivity mutations.

**Results:**

*Anopheles gambiae *GAH showed varying levels of resistance to all insecticide classes. Metabolic detoxification and reduced target-site sensitivity mechanisms were implicated. Most wild-caught families showed staggered larval time-to-hatch. However, some families were either exclusively early hatching or late hatching. Most GAH larvae hatched early but many egg batches contained a proportion of late hatching larvae. Crosses between the time-to-hatch selected sub-colonies yielded ambiguous results that did not fit any hypothetical models based on single-locus Mendelian inheritance. There was significant variation in the expression of insecticide resistance between the time-to-hatch phenotypes.

**Conclusions:**

An adaptive response to the presence of multiple insecticide classes necessarily involves the development of multiple resistance mechanisms whose effectiveness may be enhanced by intra-population variation in the expression of resistance phenotypes. The variation in the expression of insecticide resistance in association with selection for larval time-to-hatch may induce this kind of enhanced adaptive plasticity as a consequence of pleiotropy, whereby mosquitoes are able to complete their aquatic life stages in a variable breeding environment using staggered larval time-to-hatch, giving rise to an adult population with enhanced variation in the expression of insecticide resistance.

## Background

Malaria is holoendemic in Ghana and is responsible for an estimated 22% of mortality in children under five, as well as 9% of maternal deaths [1], a situation that is mirrored in much of West Africa.

The major vectors of malaria in the West African region are *Anopheles gambiae*, *Anopheles funestus *and *Anopheles arabiensis *[2]. *Anopheles gambiae *is the nominal member of the *An. gambiae *complex and is sub-divided into 5 informally named chromosomal forms [3] and two molecular forms, M and S [4]. Recent evidence suggests that the M molecular form can be further subdivided into two distinct breeding units [5]. Insecticide resistance has been detected in a large number of *An. gambiae *populations, particularly in the West African region [6-9]. The incidence of multiple resistances to insecticides is increasing and poses a threat to malaria vector control. This is because there are currently only four classes of insecticides available for use in malaria control and these collectively target only two insect neurological sites. In a mosquito survey carried out for the AngloGold Ashanti gold mine in Obuasi, Ghana, resistance to multiple insecticides was detected in *An. gambiae *and *An. funestus *[10].

Larval hatching in *An. gambiae *generally occurs 2-3 days after oviposition depending on environmental conditions [11]. However, there are descriptions of hatching occurring much later in a non-uniform way [12,13]. Yaro *et al *[12] determined the distribution of hatching time in different water types for *An. gambiae *M and S forms and showed that over 80% hatched within the first three days following oviposition regardless of water type. Between 5% and 16.8% of the total number of eggs hatched over the next four days while between 0.6% and 7.2% hatched after the first week. The S form produced significantly more hatchlings in the 3-7 day post oviposition period than the M form, and the M form showed significantly higher levels of hatching one week post-oviposition than the S form. It was also found that the type of water in which the eggs were kept had a highly significant effect on hatching with the distribution most distinct in puddle water. Yaro *et al *[12] concluded that larval time-to-hatch is determined by environmental conditions and that intraspecific variation in hatching time is an adaptation to survive variable conditions such as breeding site flooding and desiccation. They also suggest that eggs are not passive and that the time taken for an egg to hatch is probably dependent on water factors such as bacterial composition and oxygen content.

Here the adaptive significance of larval time-to-hatch is further assessed by investigating the possibility of a genetic component controlling time-to-hatch as well as the expression of insecticide resistance in association with time-to-hatch in *An. gambiae*.

## Methods

### Laboratory colony material

The GAH *An. gambiae *S form colony originating from Ghana and colonized in 2006 formed the basis of this study. SUA, an insecticide susceptible *An. gambiae *colony from Liberia, was used as the susceptible reference strain in insecticide bioassays. All mosquitoes were reared in the Botha De Meillon insectary at the National Institute for Communicable Diseases, NHLS, Johannesburg. Conditions were maintained at approximately 25°C with 75-85% relative humidity in a twelve-hour light: dark cycle with 30 min dusk and dawn transitions. Larvae were fed on ground dog biscuits and yeast and adults received three blood meals per week.

### Wild caught material

Samples of *An. gambiae *were collected from Ahafo, Ghana (7°03.656N; 2° 24.190W), in June 2008; Damang (5°30.992N; 1°52.022W) and Tarkwa (5° 22.383N; 2°01.017W), Ghana, in January 2009; Pointe Noire, Republic of the Congo (4°40'31S; 11°58'14E), in March 2009. The samples from Ghana were used for various evaluations in conjunction with laboratory-reared material as described below. The material from the Republic of the Congo served as a comparative *An. gambiae *sample from a different region.

### Field collections

Mosquitoes were collected in Ghana and the Republic of the Congo from inside human dwellings using a torch and aspirator. Mosquitoes were placed in polystyrene cups and were provided with a 10% sugar solution prior to transportation.

### Species identification

Wild-caught mosquito samples were transported to the NICD and were initially sorted using morphological keys [14,15]. Those identified as members of the *An. gambiae *complex were identified to species using the *An. gambiae *species-specific PCR assay [16]. All *An. gambiae sensu stricto *samples were further characterized as either M or S molecular form by PCR [17].

### Larval early and late hatch selections

GAH colony mosquitoes were selected at the larval stage according to early and late hatch phenotypes. During initial selections larvae from the base colony were allowed to develop for approximately 10 days, at which point all fourth instar larvae were removed and placed into a new bowl labelled GAH Early Hatch. The second and third instar larvae were returned to the baseline colony. All first instar larvae at 10 days as well as remaining unhatched eggs were pooled as GAH Late Hatch. Larvae selected in this way were reared through to adults. This process was repeated using several egg batches until separate Early and Late Hatch adult sub-colonies had been produced. These were maintained and blood-fed according to the standard procedure. Eggs from each sub-colony were harvested and the time-to-hatch selection procedure was repeated. All larvae that hatched within four days of oviposition from the Early Hatch sub-colony were kept as GAH Early Hatch while the remaining eggs were transferred to the baseline colony. The Late Hatch sub-colony was selected by transferring all larvae that hatched within four days of oviposition to the baseline colony so that all larvae that hatched subsequently remained as GAH Late Hatch. These selections continued for at least six generations before cross-mating experiments or insecticide susceptibility bioassays were conducted.

### Cross-mating experiments

In order to determine whether there is a genetic component associated with larval time-to-hatch, cross-mating experiments between the Early and Late Hatch sub-colonies were set up. Early Hatch females were crossed with Late Hatch males and vice versa. The numbers of eggs produced from each cross were quantified. Eggs were monitored for hatching and the numbers of F1 hatchlings from each cross were recorded daily. F1 larvae from each cross were reared to adults and these were either back-crossed to the parental strains or were intercrossed. Eggs and F2 hatchlings from each back-cross or intercross were monitored daily as described above. Egg production and larval hatching in the baseline GAH colony was concurrently monitored as a control.

### Determining hatch proportions of eggs from early and late hatch selected sub-colonies, cross-mating experiments and the baseline *An. gambiae *GAH colony

Eggs from each sub-colony, the baseline colony and the cross-mating experiments were collected and placed in marked egg bowls. The date of oviposition was recorded for each egg batch and the numbers of eggs quantified. Bowls were checked every morning and any larvae present were counted and removed. Hatchlings that emerged within four days of oviposition were classified as early hatch and those that emerged four days or more post-oviposition were classified as late hatch. Four days post oviposition was used as the cut-off point between early and late hatch as initial observations on the progeny of wild-caught mosquitoes showed that the majority of eggs hatch within four days while the rest tend to hatch in a staggered fashion for up to 22 days post oviposition.

### Determining larval time-to-hatch distributions in families reared from wild-caught *An. gambiae *females from Ghana and the Republic of the Congo

Blood-fed, wild-caught *An. gambiae *females from Ghana and the Republic of the Congo were individually placed in vials lined with moist filter paper for oviposition. Cotton wool pads soaked in a 10% sugar solution were provided. Vials were monitored daily for eggs. All eggs produced were placed in egg bowls by family and were monitored daily for hatching. Hatchlings were counted and removed as described for the cross-mating and time-to-hatch selection experiments. Those families reared from wild-caught females from Ahafo, Ghana, formed the pilot study from which the four-day cut off point for classifying the early and late hatch phenotypes was determined. This cut off point closely approximates with information from Yaro *et al *[12].

### Effect of egg density on hatch success and time-to-hatch in *An. gambiae*

The batches of eggs produced by each wild-caught female that was used in the hatch distribution analysis were also used to determine whether the number of eggs produced by a single female had an effect on the proportion of eggs that hatched, or on the proportion of eggs that hatched early. Data were grouped according to number of eggs laid and analysed using scatter-plots and linear regression in Statistix 7 (Analytical Software, Tallahassee, FL, USA).

### The effect of water disturbance on egg hatching in *An. gambiae *GAH

An experiment to test the effect of disturbance on egg hatching from the time-to-hatch selected sub-colonies and the baseline GAH colony was performed. Three batches (replicates) of eggs from each group were obtained and divided into two cohorts per batch for different treatments. One cohort was rinsed into a larval bowl that contained a floating plastic ring, but the eggs were not specifically contained within this ring and so could be stranded on the sides of the bowl due to water evaporation. These eggs were sprayed with water (disturbed) daily. The other cohort was also rinsed into a larval bowl so that all the eggs were contained within the floating plastic ring. This ring prevented the eggs from being stranded on the sides of the larval bowl and thus they did not require any disturbance to keep them in contact with the water. The disturbed and undisturbed egg batches were monitored daily for hatchlings, which were counted and removed. Each set of experiments lasted 25 days after which all eggs were disturbed to determine if any unhatched eggs would hatch, and later discarded. The total number of eggs that hatched as well as the proportions of early and late hatching larvae per group was determined and analysed using two-sample t-tests or ANOVA (Statistix 7).

### Insecticide susceptibility assays

In order to test for associations between insecticide resistance phenotypes and larval time-to-hatch, adult insecticide susceptibility assays were performed on samples drawn from the *An. gambiae *GAH colony as well as from the larval time-to-hatch sub-colonies. Susceptibility to four classes of insecticide was assessed (see Table 1) according to the standard WHO procedure [18]. Approximately 125 adult female mosquitoes between the ages of two and five days, divided into five replicates of 25 mosquitoes each, were exposed to diagnostic concentrations of each insecticide (Table 1) for one hour (except for fenitrothion which has an exposure period of two hours) using WHO test kits and insecticide treated filter papers. Knockdown was recorded at the end of the exposure period after which all mosquitoes were transferred to holding tubes for 24 hours during which they were provided with a 10% sugar solution. Mean percentage mortalities 24 h post exposure were recorded. Controls included exposures to untreated filter paper and treated paper efficacy was confirmed by exposing samples drawn from the insecticide susceptible reference *An. gambiae *strain, SUA, to the insecticide treated papers. Data were analysed by performing two sample t-tests using Statistix 7 (Analytical Software, Tallahassee, FL, USA).

**Table 1:** Insecticides used for adult insecticide susceptibility tests against the Anopheles gambiae laboratory colony GAH as well the larval time-to-hatch selected sub-colonies.

Insecticide class	Insecticides used (concentration)
organochlorines	dieldrin (4%); DDT (4%)

organophosphates	malathion (5%); fenitrothion (1%); pirimiphos methyl (0.9%)

carbamates	bendiocarb (0.1%); propoxur (0.1%)

pyrethroids	permethrin (0.75%); deltamethrin (0.05%)

Insecticides are listed by class with diagnostic concentrations in parentheses.

### Synergist bioassays

A set of enzyme synergists was used to test for associations between enzyme activity and the expression of insecticide resistance in the *An. gambiae *GAH laboratory colony and the time-to-hatch selected sub-colonies. Enzyme synergists can be employed in this manner because they are recognized as substrates by those enzyme systems implicated in insecticide detoxification [19].

The synergists used were 20% diethyl maleate (DEM), 10% triphenylphosphate (TPP) and 4% piperonyl butoxide (PBO). These generally synergize glutathione S-transferase (GST), esterase and monooxygenase enzyme activity respectively. However, the specificity of each synergist is likely to be affected by the metabolic pathways associated with resistance and cross-reaction can occur. Approximately 25 adult female mosquitoes, 2 to 5 days old, were exposed to synergist for one hour immediately followed by exposure to a diagnostic concentration of insecticide for 1 h (2 h for fenitrothion). A similar sample was concurrently exposed to insecticide only for 1 h. Controls included concurrent exposure to synergist only as well as exposure to untreated paper. Mortalities were recorded 24 h post exposure. At least five replicates per synergist for each insecticide were performed. Mosquitoes that survived synergist bioassays, as well as those that died following insecticide exposure only, were collected and stored on silica before undergoing molecular screening for target site mutations associated with insecticide resistance. Final mortalities from the synergized samples were compared to their corresponding unsynergized samples using two sample t-tests (Statistix 7).

### Screening for reduced target site sensitivity mutations

DNA was extracted from samples to be used in molecular assays according to the method of Collins *et al *[20] or using a ZyGEM PrepGEM™ insect (ZyGEM Corp Ltd.) extraction kit following the supplied protocol. Individual mosquitoes were screened for *kdr*, *ace-1^R ^*and *Rdl *genotypes. Data were analysed using Fisher's Exact test, χ^2^, or two sample t-tests (Statistix7).

### Hydrolysis probe molecular assays

The *kdr *and *Rdl *TaqMan^® ^hydrolysis probe molecular assays [21,22] were used to detect *kdr *(L1014F and L1014S) and *Rdl *mutations associated with resistance to pyrethroids and DDT (*kdr*) and resistance to dieldrin (*Rdl*). All primers used in the real time experiments were supplied by Inqaba Biotechnical Industries, Hatfield, Pretoria, and probes were supplied by Applied Biosystems Inc, Forster City, CA, USA. *Kdr *and *Rdl *homozygous (RR and SS) and heterozygous (RS) positive controls as well as no template controls were included in all assays.

### Restriction fragment length polymorphism assay to detect the *ace-1^R ^*mutation

The *ace-1^R ^*mutation in acetylcholinesterase 1 has previously been associated with organophosphate and carbamate resistance in *An. gambiae *[23]. DNA was extracted from samples that survived exposure to 0.1% bendiocarb as well as from samples that died following exposure. The *Alu*1 restriction enzyme (Roche Diagnostics, Basel, Switzerland) digest was used to genotype samples for *ace-1^R ^*[23].

## Results

### Species identification of wild-caught and colony samples

All wild-caught mosquitoes used in the time-to-hatch experiments were identified as *An. gambiae s.s*. Of the Ghana Ahafo families, two (Gahf 33 and 49) were M form whilst the remaining 26 were S form. All Republic of the Congo (COGS, N = 18) and Ghana Damang and Tarkwa (Ghag, N = 5) families were S form. The GAH laboratory colony was confirmed as S form.

### Larval time-to-hatch distributions of wild-caught families

**Figure 1:**
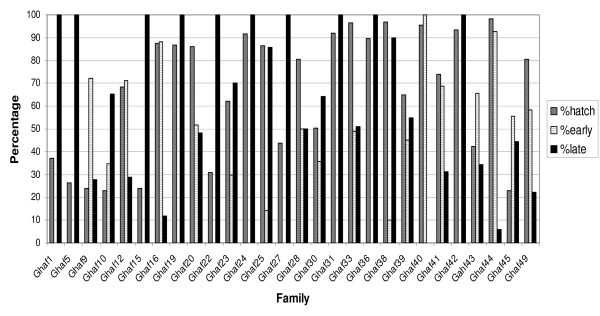
***Anopheles gambiae *families from Ahafo, Ghana**. Overall larval hatch rate per family (percentage of eggs that hatched) and the proportions of larvae that hatched either early or late using three days post oviposition as the cut-off between time-to-hatch phenotypes are shown.

Most Ghana Ahafo families (Gahf) showed bimodal hatching. Only one family (Gahf 41) was entirely early hatching (3.6%) and ten families (Gahf 1, 9, 15, 19, 22, 24, 27, 31, 35, 42) were entirely late hatching (35.7%) (Figure 1). In this experiment, three days post oviposition was used as the cut-off between the early and late hatch phenotypes. The cut-off point was shifted to four days for all subsequent experiments as the majority of the larvae hatched within this period following which hatch proportions dropped off significantly.

**Figure 2:**
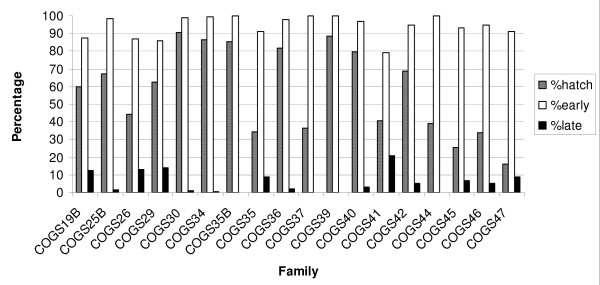
***Anopheles gambiae *families from Pointe Noire, Republic of Congo**. The overall larval hatch rate per family (percentage of eggs that hatched) and the proportions of larvae that hatched either early or late using four days post oviposition as the cut-off between time-to-hatch phenotypes are shown.

Most families reared from wild-caught females from the Republic of the Congo (COGS) showed bimodal hatching. Four (22.22%) were entirely early hatching and none were entirely late hatching using four days post oviposition as the cut-off point between time-to-hatch phenotypes (Figure 2).

**Figure 3:**
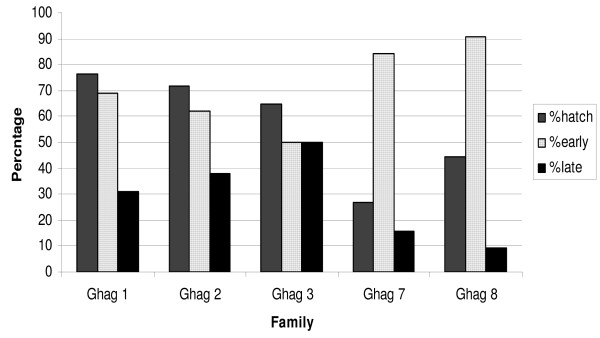
***Anopheles gambiae *families from Damang and Tarkwa, Ghana**. Overall larval hatch rate per family (percentage of eggs that hatched) and the proportions of larvae that hatched either early or late using four days post oviposition as the cut-off between time-to-hatch phenotypes are shown.

All Ghana Damang and Tarkwa (Ghag) families showed bimodal hatching. The proportions of late hatching larvae per family (Figure 3) were generally higher than those observed in the COGS families.

### Larval time-to-hatch proportions in laboratory colonies and progeny of cross-mating experiments

**Figure 4:**
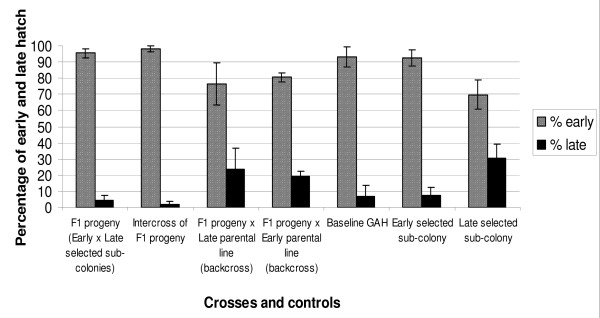
**Proportions of early and late hatching *Anopheles gambiae *larvae per cross, GAH laboratory colony (baseline) and time-to-hatch selected (Early and Late) sub-colonies**.

The overall proportions of F1 and F2 progeny that hatched from the crosses, back-crosses and intercross as well as progeny from the unselected GAH colony and time-to-hatch selected sub-colonies did not vary significantly (ANOVA, P > 0.05). Between 400 and 3000 eggs per cross or selected sub-colony were monitored in total. The latest hatch occurred 22 days post oviposition, but in general hatching after 9 days was uncommon. The vast majority of larvae hatched early across all batches (Figure 4). The highest proportions of late hatching larvae were recorded in the Late Hatch selected sub-colony and in the F2 progeny of both back-crosses to the parental sub-colonies. These late hatching proportions were numerically higher than those recorded in the GAH laboratory colony, the Early Hatch selected sub-colony, the F1 hybrid progeny of Early Hatch crossed with Late Hatch and F2 progeny of the hybrid intercross. However, these differences were not significant for untransformed data (ANOVA: P > 0.1), and were only significant at lower confidence for arcsine-transformed data (P = 0.08). In summary, these data do not correlate with any hypothetical models based on Mendelian inheritance of a single genetic factor controlling larval time-to-hatch, but suggest that larval time-to-hatch phenotypes can be selected for (Figure 4).

### Effect of egg density on hatching

Linear regressions were performed on each group of wild-caught families (Gahf, Ghag and COGS) to test for an association between the number of eggs laid per female and the proportion of eggs that hatched, or the number of eggs laid per female and the proportion of eggs that hatched early. No significant trends were detected (P > 0.05) suggesting that the number of eggs laid by a female does not influence the proportion that hatch or the proportions of eggs that hatch early.

### Effect of water disturbance on egg hatching

The total numbers of eggs monitored ranged from 1,600 to 3,200 per experiment, divided between three replicates per experiment. Significantly fewer eggs from the undisturbed experiment hatched compared to the disturbed experiment, indicating that egg disturbance is necessary for optimal hatching (two sample t-tests: P < 0.01 for GAH baseline; P = 0.01 for the Early Hatch sub-colony; P < 0.01 for the Late Hatch sub-colony) (Table 2). In addition, there was a significant difference in the total percentage of eggs that hatched in the undisturbed experiment between the Early and Late Hatch selected sub-colonies, and between the Early Hatch selected sub-colony and the baseline colony (two sample t-tests: P < 0.01 and P = 0.01, respectively) with significantly higher proportions of hatching occurring in the Early Hatch sub-colony. No significant differences in total hatch were observed between groups for the disturbed experiment. There were significantly higher proportions of late hatching larvae in the Late Hatch selected sub-colony compared to the Early Hatch selected sub-colony and vice versa regardless of whether the eggs were disturbed or not (two-by-two contingency tables: P < 0.05 in all cases) (Table 2), showing significant assortment of hatching phenotype with time-to-hatch sub-colony selection.

**Table 2:** Mean egg hatch proportions as well as late and early hatch proportions in unselected and time-to-hatch selected sub-colonies of Anopheles gambiae GAH.

Colony/sub-colony	Hatch rate/time-to-hatch proportion	Disturbed (%)	Undisturbed (%)
GAH base	Mean hatch	78	20.4
	Proportion late	20.15	29.87
	Proportion early	79.85	70.13

Early Hatch	Mean hatch	75	47.23
	Proportion late	5	25.4
	Proportion early	95	74.6

Late Hatch	Mean hatch	90.2	11.4
	Proportion late	19.9	37.8
	Proportion early	80.1	62.2

Data are given as mean percentages and are sorted according to whether or not eggs were disturbed prior to hatching.

### Insecticide susceptibility assays

**Figure 5:**
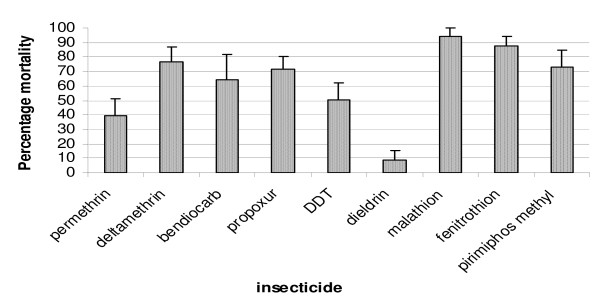
**Mean percentage mortalities of 2-5 day old *Anopheles gambiae *laboratory colony (GAH) females 24 h post exposure to listed insecticides**. Assays were based on the standard WHO bioassay method for testing adult susceptibility to insecticides (WHO, 1998).

Based on WHO criteria [18], *An. gambiae *GAH showed various levels of resistance to permethrin and deltamethrin (pyrethroids), bendiocarb and propoxur (carbamates), DDT and dieldrin (organochlorines) and pirimiphos methyl (organophosphate). Resistance to the organophosphates fenitrothion and malathion is also suggested (Figure 5).

**Figure 6:**
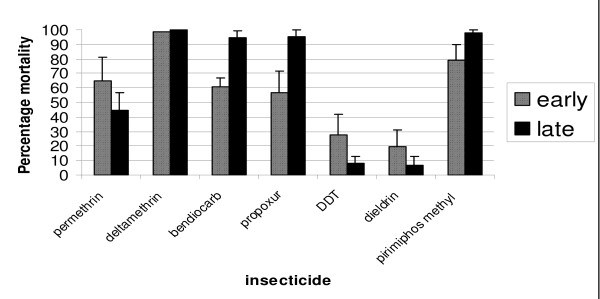
**Mean percentage mortalities of 2-5 day old *Anopheles gambiae *time-to-hatch selected sub-colony (GAH) females 24 h post exposure to listed insecticides**. Assays were based on the standard WHO bioassay method for testing adult susceptibility to insecticides (WHO, 1998).

The larval time-to-hatch selected sub-colonies differed significantly from each other through all insecticide resistance phenotypes with the exception of the pyrethroid deltamethrin against which neither sub-colony showed resistance (Figure 6). The Early Hatch selected sub-colony showed significantly higher levels of resistance to the carbamates bendiocarb and propoxur as well as to the organophosphate pirimiphos methyl than the Late Hatch selected sub-colony (two sample t-tests: P < 0.01 in all cases). This trend was reversed in response to exposure to DDT, dieldrin and permethrin, with the Late Hatch selected sub-colony showing significantly higher levels of resistance (two sample t-tests: P < 0.01 in all cases).

### Synergist bioassays

Pyrethroid resistance was significantly synergized by PBO and TPP in *An. gambiae *GAH. These data implicate esterases and P450 monooxygenases in pyrethroid metabolism in Ghanaian *An. gambiae*. No other insecticide resistance phenotypes reduced in expression following exposure to synergists (Table 3).

**Table 3:** Mean percentage mortalities of PBO, TPP or DEM synergized and unsynergized samples of 2-5 day old Anopheles gambiae laboratory colony (GAH) females 24 h post exposure to listed insecticides (syn = synergized; unsyn = unsynergized. Bolded p-values are significant at 95% confidence intervals).

Synergist		4% PBO		10% TPP		20% DEM	
**Insecticide**	**Syn/unsyn**	**mean % mortality**	**t-test p**	**mean % mortality**	**t-test p**	**mean % mortality**	**t-test p**

4% DDT	syn	27.46	0.99	39.6	0.76	38.37	0.86
	unsyn	27.48		41.8		37.38	

4% dieldrin	syn	11.70	0.81	13.8	0.13	12.15	0.89
	unsyn	10.83		9.9		12.65	

permethrin	syn	80.54	** < 0.01**	86.14	** < 0.01**	74.93	0.16
	unsyn	48.72		56.11		61.78	

deltamethrin	syn	95.32	** < 0.01**	94.12	0.98	100	**~**1
	unsyn	72.52		94.60		98	

propoxur	syn	70.30	0.87	84.98	0.06	79.43	0.52
	unsyn	71.03		79.5		82.23	

bendiocarb	syn	77.74		78.8	0.16	68.99	0.66
	unsyn	71.56	0.29	71.36		72.43	

pirimiphos methyl	syn	33.39	0.37	86.65	0.08	74.65	0.09
	unsyn	44.89		67.56		63.53	

bendiocarb	syn	77.74		78.8	0.16	68.99	0.66
	unsyn	71.56	0.29	71.36		72.43	

pirimiphos methyl	syn	33.39	0.37	86.65	0.08	74.65	0.09
	unsyn	44.89		67.56		63.53	

Pyrethroid resistance was significantly synergized by PBO in the Late Hatch selected sub-colony (two sample t-test: P = 0.02). This effect was not detected in the Early Hatch selected sub-colony as resistance to pyrethroids had diminished by this generation with over 90% of the Early Hatch selected mosquitoes not surviving permethrin exposure. TPP and PBO induced no significant reductions in insecticide resistance expression in the time-to-hatch selected sub-colonies when challenged against the other insecticides listed in table 3. DEM was not assayed against the time-to-hatch selected sub-colonies as it induced no effect on insecticide resistance in the GAH baseline colony.

### Screening for reduced target site sensitivity mutations

#### *kdr*

**Figure 7:**
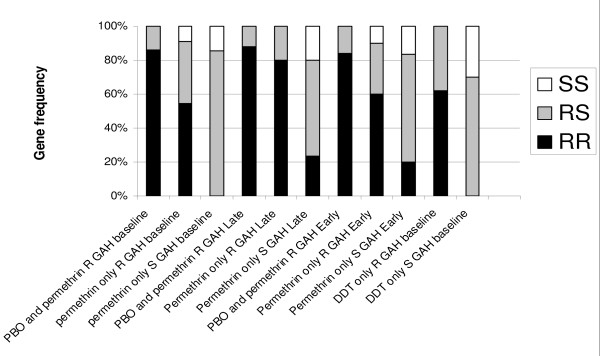
**Proportions of L1014F *kdr *genotypes sorted by response to insecticide exposure phenotype (resistant or susceptible)**. All samples were comprised of female *Anopheles gambiae *laboratory reared mosquitoes from either the GAH baseline colony or the time-to-hatch selected sub-colonies exposed to permethrin only, permethrin and PBO, or DDT only. R = resistant, S = susceptible.

Samples genotyped for the L1014F *kdr *mutation were drawn from the GAH laboratory colony and the larval time-to-hatch selected sub-colonies. Sample sizes ranged from 18 to 30. All mosquitoes assayed were characterized by insecticide exposure bioassay as either insecticide resistant or susceptible. Some were further characterized by their response to insecticide exposure following pre-exposure to PBO. There was significant variation in *kdr *allele frequency between resistant and susceptible samples (Figure 7) whereby clear associations between *kdr *and permethrin resistance (GAH baseline χ^2 ^= 18.47, P < 0.01; Late Hatch χ^2 ^= 35.07, P < 0.01; Early Hatch χ^2 ^= 11.41, P < 0.01) as well as *kdr *and DDT resistance (χ^2 ^= 43.43, P < 0.01) were detected. However, several homozygous resistant RR genotypes were recorded in the permethrin susceptible samples and a small proportion of homozygous susceptible SS genotypes were recorded in the permethrin resistant samples. Pre-exposure to PBO significantly increased the *kdr *frequency amongst permethrin resistant samples in the GAH baseline colony (χ^2 ^= 15.01, P < 0.01) and the GAH Early Hatch sub-colony (χ^2 ^= 10.49, P < 0.01) but not in the GAH Late Hatch sub-colony (χ^2 ^= 0.24, P > 0.05).

The hydrolysis probe molecular assay [21] was also used to genotype the L1014S *kdr *mutation in 82 permethrin and DDT exposed GAH females. All were genotyped as homozygous susceptible SS.

#### *Rdl*

A sample of *An. gambiae *GAH females were characterized as either dieldrin resistant or susceptible following exposure to 4% dieldrin. The alanine296-glycine (*Rdl*) GABA receptor mutation was detected by hydrolysis probe assay [22] and there was a clear association between *Rdl *genotype and response to dieldrin phenotype in both the time-to-hatch selected sub-colonies (Early Hatch χ^2 ^= 67.89, P < 0.01; Late Hatch χ^2 ^= 108, P < 0.01) as well as the base colony (χ^2 ^= 52.06, P < 0.01) (Table 4). In addition, GAH Late survivors showed significantly higher frequencies of the *Rdl *mutation than GAH Early survivors (χ^2 ^= 5.21, P < 0.05). Exposure to PBO prior to dieldrin exposure did not increase mortality in GAH (two sample t test: P = 0.81) (Table 3), as was previously observed in another *An. gambiae *colony [24], suggesting that P450 monooxygenases do not play a significant role in resistance to dieldrin in GAH.

**Table 4:** Numbers and proportions of alanine296-glycine (Rdl) Anopheles gambiae GAH female genotypes sorted according to response to dieldrin exposure phenotype (resistant or susceptible).

	GAH base Susceptible	GAH base Resistant	GAH Late Susceptible	GAH Late Resistant	GAH Early Susceptible	GAH Early Resistant
**Genotype**	**Frequency**	**Frequency**	**Frequency**	**Frequency**	**Frequency**	**Frequency**

**SS**	23/25 = 92%	0	23/25 = 92%	0	24/27 = 89%	0

**RS**	2/25 = 8%	16/29 = 55.17%	2/25 = 8%	12/25 = 48%	3/27 = 11%	17/22 = 77%

**RR**	0	13/29 = 44.83%	0	13/25 = 52%	0	5/22 = 23%

S = dieldrin susceptible *Rdl*^+^, R = dieldrin resistant *Rdl*.

#### *ace-1^R^*

Twenty-eight bendiocarb exposed *An. gambiae *GAH female survivors and 27 bendiocarb exposed females that died following exposure were screened for the *ace-1^R ^*mutation using the RFLP assay [18]. Three of the 28 survivors were scored as homozygous resistant and the remaining 25 survivors were scored as heterozygous. All 27 mosquitoes that died following exposure to bendiocarb were scored as homozygous susceptible. These data show a strong association between *ace-1^R ^*genotype and response to bendiocarb exposure phenotype (χ^2 ^= 180; P < 0.01). The apparent scarcity of the RR (homozygous resistant) genotype may be attributable to an unusually high death rate of RR individuals at pupation as previously described [25].

## Discussion

Resistance to multiple classes of insecticides in *An. gambiae *populations is a growing concern for malaria vector control [6]. The *An. gambiae *laboratory colony GAH shows resistance to all classes of insecticide currently available for use in public health. Although this colony is likely to show reduced genetic variation relative to the wild population from which it is derived, multiple insecticide resistance in *An. gambiae *has previously been reported from Ghana [10].

From the data presented here, mechanisms of resistance to pyrethroids in *An. gambiae *GAH include monooxygenase and esterase mediated detoxification coupled with the L1014F *kdr *mutation. The correlation between *kdr *genotype and pyrethroid resistance phenotype was clear but not absolute, with small numbers of homozygous resistant (RR) genotypes occurring in the phenotypically susceptible samples and vice versa. These discrepancies, coupled with the occurrence of RS heterozygotes in resistant and susceptible mosquitoes as well as an increase in *kdr *frequency in PBO synergized samples, suggest that enzyme mediated detoxification also plays an important role in the production of a measurable pyrethroid resistance phenotype [26,27]. Resistance to DDT associated particularly closely with the assortment of L1014F *kdr*. Resistance to dieldrin associated closely with the *Rdl *mutation while resistance to the carbamate bendiocarb associated closely with the *ace-1*^*R *^mutation. Carbamate resistance and the low level of organophosphate resistance recorded in the *An. gambiae *GAH colony is therefore likely mediated by the assortment of *ace-1*^*R *^although enzyme mediated detoxification may play a supporting role. High frequencies of *ace-1^R ^*have been recorded in *An. gambiae *S form populations in Burkina Faso indicating that caution should be employed when using carbamate and organophosphate insecticides for vector control programs in the West African region [28].

The majority of *An. gambiae *GAH eggs hatched within four days after oviposition. However, the proportion of late hatching eggs increased following selection for this phenotype, suggesting that, in addition to environmental factors [12], there may be a genetic component controlling larval time-to-hatch. Cross-mating between the time-to-hatch phenotypes, supported by hybrid inter-crosses and back-crossing of hybrids to the parental strains, gave ambiguous results suggesting that this genetic component may be multi-factorial. A genetic influence on variation in larval time-to-hatch is further supported by the observation that some of the wild-caught *An. gambiae *families from Ghana and the Republic of the Congo were exclusively early or late hatching. Importantly, there was also significant variation in the expression of insecticide resistance between the time-to-hatch phenotypes. The variation recorded in the response to insecticide exposure assays also correlates with variation in *kdr *and *Rdl *frequencies between the time-to-hatch selected sub-colonies, showing that selection for time-to-hatch inadvertently affected the frequencies of those factors controlling insecticide resistance.

Although the adaptive significance of staggered larval time-to-hatch post oviposition has not been quantified, it is highly likely that this characteristic has evolved in response to the variable nature of preferred *An. gambiae *breeding sites [12]. These sites are typically small, are highly variable in terms of water quality, are subject to large fluctuations in temperature and are susceptible to desiccation and flooding. That variation in larval time-to-hatch is ubiquitous across *An. gambiae *populations is supported by the quantification of this characteristic in geographically diverse samples from the Republic of the Congo and Ghana.

These data further show that the number of eggs that hatch in total as well as the proportions of eggs that hatch either early or late do not depend on the initial number of eggs laid per *An. gambiae *female. This suggests that delayed larval hatch is not an adaptation to avoid over-crowding or competition for resources. Additionally, water disturbance induces a significantly higher rate of egg hatching in *An. gambiae*.

## Conclusions

The presence of insecticides in fluctuating concentrations significantly alters the chemical environment in which mosquitoes breed and rest. A sufficient adaptive response to the presence of multiple insecticide classes necessarily involves the development of multiple resistance mechanisms whose effectiveness may be enhanced by intra-population variation in the expression of resistant phenotypes. The variation in the expression of insecticide resistance in association with selection for larval time-to-hatch described here may induce this kind of enhanced adaptive plasticity as a consequence of pleiotropy. In this scheme cohorts of mosquitoes are able to complete their aquatic life stages in a variable breeding environment using staggered larval time-to-hatch, giving rise to an adult population with enhanced variation in the expression of insecticide resistance. Enhanced variation inadvertently produced in this way offers a wider platform for the continued development of resistance to insecticides.

Successfully managing multiple insecticide resistance in malaria vector control should involve an appraisal of the biological and adaptive variation inherent within target vector populations. Effective malaria control can then be achieved by adopting an evidence-based approach, which incorporates the principles of judicious insecticide use in a broader Integrated Vector Management (IVM) system.

## Competing interests

The authors declare that they have no competing interests.

## Authors' contributions

MLK conducted all the experiments, analysed the results and drafted the manuscript; LLK assisted in the design and analysis of the molecular experiments and drafting of the manuscript; MC and RHH assisted with the design of the project and the drafting of the manuscript; BDB designed the project, assisted with data interpretation and statistical analysis and completed the drafting of the manuscript. All field collections were conducted under guidance of RHH. All authors read and approved the final manuscript.
